# HIV Incidence and Risk Factors for Acquisition in HIV Discordant Couples in Masaka, Uganda: An HIV Vaccine Preparedness Study

**DOI:** 10.1371/journal.pone.0024037

**Published:** 2011-08-31

**Authors:** Eugene Ruzagira, Symon Wandiembe, Andrew Abaasa, Agnes N. Bwanika, Ubaldo Bahemuka, Pauli Amornkul, Matthew A. Price, Heiner Grosskurth, Anatoli Kamali

**Affiliations:** 1 Medical Research Council (MRC)/Uganda Virus Research Institute (UVRI) Uganda Research Unit on AIDS, Entebbe, Uganda; 2 International AIDS Vaccine Initiative, New York, New York, United States of America; 3 London School of Hygiene and Tropical Medicine, London, United Kingdom; University of KwaZulu-Natal, South Africa

## Abstract

**Objectives:**

To determine the incidence of and risk factors for HIV acquisition in a cohort of HIV-uninfected partners from HIV discordant couples in Masaka, Uganda, and to establish its suitability for HIV vaccine trials.

**Methods:**

HIV-uninfected adults living in HIV discordant couple relationships were enrolled and followed for 2 years. Interviews, medical investigations, HIV counseling and testing, syphilis and urine pregnancy (women) tests were performed at quarterly visits. Sexual risk behaviour data were collected every 6 months.

**Results:**

495 participants were enrolled, of whom 34 seroconverted during 786.6 person-years of observation (PYO). The overall HIV incidence rate [95% confidence interval (CI)] was 4.3 [3.1–6]; and 4.3 [2.8–6.4] and 4.4 [2.5–8] per 100 PYO in men and women respectively. Independent baseline predictors for HIV acquisition were young age [18–24 (aRR = 4.1, 95% CI 1.6–10.8) and 25–34 (aRR = 2.7, 95% CI 1.2–5.8) years]; alcohol use (aRR = 2.6, 95% CI 1.1–6); and reported genital discharge (aRR = 3.4, 95% CI 1.6–7.2) in the past year. Condom use frequency in the year preceding enrolment was predictive of a reduced risk of HIV acquisition [sometimes (aRR = 0.4, 95% CI 0.2–0.8); always (aRR = 0.1, 95% CI 0.02–0.9)]. In the follow-up risk analysis, young age [18–24 (aRR = 6.2, 95% CI 2.2–17.3) and 25-34 (aRR = 2.3, 95% CI 1.1–5.0) years], reported genital discharge (aRR = 2.5, 95% CI 1.1–5.5), serological syphilis (aRR 3.2, 95% CI 1.3–7.7) and the partner being ART naïve (aRR = 4.8, 95% CI 1.4–16.0) were independently associated with HIV acquisition. There were no seroconversions among participants who reported consistent condom use during the study.

**Conclusions:**

The study has identified important risk factors for HIV acquisition among HIV discordant couples. HIV-uninfected partners in discordant couples may be a suitable population for HIV vaccine efficacy trials. However, recent confirmation that ART reduces heterosexual HIV transmission may make it unfeasible to conduct HIV prevention trials in this population.

## Introduction

HIV/AIDS continues to be a significant global health problem. Sub-Saharan Africa remains the most affected region with heterosexual intercourse being the main mode of HIV transmission [1]. A large proportion of new HIV infections in sub-Saharan Africa occur within stable HIV discordant heterosexual couples [2], [3], [4], [5], [6], and, HIV discordance is highly prevalent in diverse populations [7], [8].

The high prevalence of HIV discordance and the high rates of HIV transmission within discordant couples make them a potentially suitable population for clinical trials evaluating preventive vaccines, microbicides, pre-exposure prophylaxis and other HIV prevention interventions in Africa [6], [8]. Before such trials are conducted, preparatory studies are needed to estimate HIV incidence and assess feasibility of recruiting and retaining volunteers for the duration of a trial [9]. Such data are also required to support trial design and sample size estimation and the selection of the most appropriate populations [10].

We report the incidence of and risk factors for HIV acquisition in a cohort of HIV-uninfected partners from HIV discordant couples enrolled in an HIV vaccine trial feasibility study. The study was part of a set of HIV vaccine preparedness studies (VPS) conducted by the Medical Research Council (MRC)/Uganda Virus Research Institute (UVRI) Uganda Research Unit on AIDS in collaboration with the International AIDS Vaccine Initiative (IAVI) in Masaka district, Uganda.

## Methods

### Ethics statement

The VPS study protocol was reviewed and approved by the Science and Ethics Committee of the Uganda Virus Research Institute and by the Uganda National Council for Science and Technology. Couples were given the informed consent document to read or, if illiterate, it was read to them by a study nurse in the presence of an independent witness. The study nurse answered any questions raised by the couple before obtaining written informed consent from the HIV-uninfected partner. Later in the study, written informed consent was also obtained from HIV-infected partners to enable collection of data on receipt of antiretroviral therapy (ART). Informed consent discussions were repeated during follow up visits with both partners to ensure continued understanding of the study. Free condoms were offered throughout the duration of the study. Counseling on male medical circumcision was initiated later on in the study after evidence that this intervention was effective became available. Participants who wished to be circumcised were referred to hospitals with surgical facilities in Masaka district. Couples were also provided with free outpatient medical care including diagnosis and treatment of RTIs. HIV-infected partners were given daily trimethoprim-sulphamethoxazole for prophylaxis against opportunistic infections and had CD4/CD8 T-cell counts measured at study entry and every 6 months throughout the study. Consistent with the 2003 national ART guidelines [11], HIV-infected partners whose CD4 cell counts fell below 200/mm^3^ and those who developed an AIDS-defining illness were referred to and initiated ART at no cost at specialised centres in Masaka. Referrals were also made for other HIV related care and prevention of mother-to-child HIV transmission (PMTCT) services. Seroconverters were also referred to receive support and care, and offered participation in an ongoing acute HIV infection study.

### Study population

Study participants were HIV-uninfected individuals living in discordant couple relationships (one spouse being HIV-uninfected and the other HIV-infected). Couples suspected or already confirmed to be HIV discordant were referred for screening from three sources: (i) the MRC/UVRI home-based HIV voluntary counseling and testing (VCT) service, (ii) the MRC/UVRI clinic-based HIV VCT service, and (iii) major HIV care and VCT providers in Masaka district.

Enrollment was offered to healthy HIV-uninfected individuals aged 18–60 years, who were married to or cohabiting with an HIV-infected sexual partner, willing to give informed consent and provide locator information, be followed for 2 years, complete interviewer administered questionnaires on HIV risk factors, undergo repeated HIV counseling and testing, accept to receive and share their HIV results with their partner, and for females to be tested for pregnancy every 3 months.

### Study procedures

Screening procedures consisted of: provision of study information to the couples, obtaining informed consent from the HIV-uninfected partners, couples' HIV voluntary counseling and testing (CVCT), and study eligibility assessment. Upon enrolment, demographic and sexual risk data including data on reported symptoms of reproductive tract infections (RTIs), extramarital sex, condom use frequency and alcohol consumption were collected using a structured questionnaire. A medical history was collected and a complete physical examination performed. Venous blood was drawn for HIV and syphilis serology. Genital examinations were only conducted on participants who reported symptoms of RTIs. Data collection on receipt of antiretroviral therapy (ART) by the HIV-infected partners was initiated later in the study. Detailed locator information including telephone numbers for those who owned or had access to a phone was collected. Participants were issued with study identification cards on which scheduled study visit dates were recorded. Costs for time and travel were reimbursed.

Couples were followed at 3-month intervals for 2 years. At each follow up visit, CVCT was performed. On this occasion, study participants were asked about their recent medical history, and a symptom directed physical examination was performed. Specimens were collected and laboratory tests conducted as during the enrolment visit. Sexual risk behavior data were collected every 6 months. Interim visits were conducted for: (i) administrative reasons; (ii) HIV VCT for presumed exposure to HIV; (iii) obtaining laboratory test results from previous visits; (iv) assessment and treatment of illnesses; (v) and other reasons as requested by the participant and in response to tracing efforts. Locator information was updated as necessary.

Participants who missed study visits were reminded to come for follow up within 2 days of the missed visit by a community mobiliser. At least 2 reminder visits were conducted for each missed visit. Participants who failed to attend follow up visits without clear reason despite the reminders, and those that could not be traced were considered lost to follow up. Participants who reported separation from or death of the HIV-infected partner were withdrawn from study as soon as this information became available.

### Laboratory methods

Determine (Abbot Laboratories, Japan) rapid test was used to screen for HIV antibodies. All positive test results were confirmed by two parallel ELISAs (Vironostika Uni-Form II Ag/Ab, BioMerieux, Netherlands and Murex HIV.2.O, Murex Biotech Ltd., UK). Western blot (Genetic Systems, Biorad Laboratories) was used to resolve discrepant ELISA results. HIV testing was repeated after 2–4 weeks on a fresh specimen if the Western blot test was indeterminate. Sero-syphilis testing was done using the rapid plasma reagin (RPR) test (Biotec). RPR reactive specimens were confirmed with the Treponema pallidum Haemaglutination (TPHA) assay (Biotec). Participants were considered to have serological syphilis infection if they tested positive for TPHA and RPR with RPR titres of 1∶4 or greater. ßhCG reagent strips (Bayer Multistix 10SG) were used for urine pregnancy testing.

### Statistical analysis

Data were recorded in MS Access and analysed in Stata 10 (StataCorp, College Station, Texas, USA). All participants who completed at least one follow-up visit were eligible for statistical analysis. Person-years of observation (PYO) were calculated as the sum of the time from enrolment (baseline) to the date of the last HIV-uninfected result, or to the estimated date of HIV infection for each participant. Date of HIV infection was imputed as the mid-point of the interval between the last HIV-uninfected and the first HIV-infected result dates. Rate ratios and 95% confidence intervals were obtained for potential risk factors using a Poisson regression model. The purposeful selection algorithm [12] was used for model building. All factors for which the association attained statistical significance (P<0.1) at univariable analysis were considered for a multivariable Poisson regression model. Sex, age and male circumcision were included as *a priori* confounders. Factors remaining significant at P<0.05 were retained in the final model. Two models were fitted; a model for risk data collected at the enrolment visit (baseline risk analysis) and a more comprehensive model that included data collected at all study visits including the enrolment visit (follow-up risk analysis). In the follow-up risk analysis model, participants who reported or were diagnosed with a potential risk factor at any visit during the period under observation (but before HIV infection) were compared to those that did not have that particular risk factor throughout the study. For the purpose of identifying the effect of male circumcision, circumcision status and gender were combined into a composite variable with circumcised male, uncircumcised male and female as its categories. A separate analysis based on Turnbull's exact interval censored maximum likelihood method [13] as opposed to the mid-point imputation analysis described above was also performed.

## Results

### Enrolment, baseline characteristics and retention

Screening and enrolment were conducted from March 2006 to May 2007; quarterly follow up visits were completed in March 2009.

**Figure 1:**
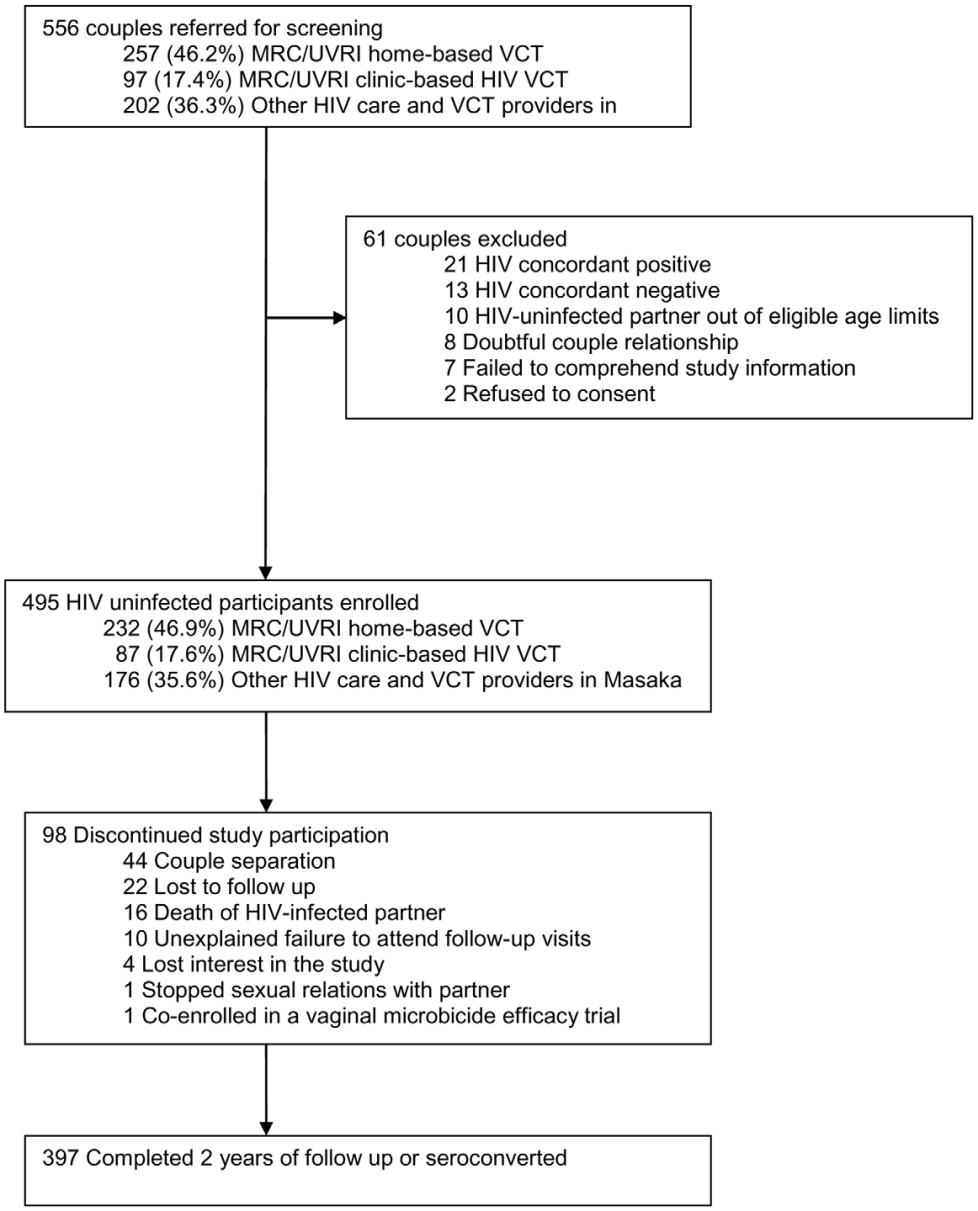
Study profile.

The study profile is shown in Figure 1. A total of 556 couples identified as discordant were screened for eligibility: 495 couples (89%) were confirmed to be HIV discordant and the HIV-uninfected partner was enrolled into the study, 27 couples (4.9%) were HIV discordant but the HIV-uninfected partner did not meet one or more of the other eligibility criteria, 21 (3.8%) were HIV concordant positive and 13 (2.3%) were HIV concordant negative. The mean age of the study participants was 36.2 years (SD: 9.2), most (69%) were male, 83% were married and only about 20% had attained some secondary school education. Of those enrolled, 482 (97.4%) participants completed at least one follow up visit while 397 (80.2%) completed 2 years of follow up or reached the study end point. Compared to those that completed the study, non-completers were younger (median age of 33 vs. 36; p<0.001), but were otherwise similar in terms of other characteristics.

### HIV incidence and baseline risk factors for HIV acquisition

Thirty-four participants seroconverted over 786.6 PYO. The overall HIV incidence rate [95% confidence interval] was 4.3 [3.1–6]; and 4.3 [2.8–6.4] and 4.4 [2.5–8] per 100 PYO in men and women respectively. Crude and adjusted associations between risk factors present at baseline and HIV incidence are presented in Table 1. Factors significantly associated with risk of HIV acquisition at univariate analysis were young age [18–24 (RR = 4.1, 95% CI 1.6–10.7) and 25–34 (RR = 2.4, 95% CI 1.1–5.2) years], alcohol use (RR = 2.6, 95% CI 1.2–5.8), condom use frequency [sometimes (RR = 0.4, 95% CI 0.2–0.8); always (RR = 0.1, 95% CI 0.01–0.7)], and reported genital discharge in the past year (RR = 3.1, 95% CI 1.6–6.1). Although not significant, the risk of HIV acquisition was higher among participants that were uncircumcised (men), not married but with steady partner, reported genital ulcer disease in the past year, had serological syphilis, and those whose HIV-infected partners were ART naïve.

**Table 1:** Baseline factors associated with incident HIV infection among HIV-uninfected partners in HIV discordant couples, Masaka, Uganda.

	N (%)	Seroconverters	PYO	Rate/ 100 PYO	RR (95% CI)	LRT p-value	aRR (95% CI)
**Total**	482 (100.0)	34	786.6	4.3			
**Male circumcision**							
Circumcised	93 (19.3)	3	152.5	2.0	1.0	0.21	1.0
Uncircumcised	237 (49.2)	20	386.4	5.2	2.6 (0.8-8.9)		1.6 (0.4-5.7)
Female	152 (31.5)	11	247.7	4.4	2.3 (0.6-8.1)		1.3 (0.3-4.9)
**Age group**							
≥35	260 (53.9)	11	447.9	2.5	1.0	0.01	1.0
25-34	175 (36.3)	16	269.1	5.9	2.4 (1.1-5.2)		2.7 (1.2-5.8)
18-24	47 (9.8)	7	69.6	10.1	4.1 (1.6-10.7)		4.1 (1.6-10.8)
**Marital status**							
Married	396 (82.2)	25	646.5	3.9	1.0	0.21	
Steady partner	86 (17.8)	9	140.1	6.4	1.7 (0.8-3.6)		
**Religious affiliation**							
Non Muslim	383 (79.5)	30	622.6	4.8	1.0	0.16	
Muslim	99 (20.5)	4	164.0	2.4	0.5 (0.2-1.4)		
**Education level**							
≥Secondary	94 (19.5)	6	151.4	4.0	1.0	0.93	
Primary	338 (70.1)	25	555.2	4.5	1.1 (0.5-2.8)		
None	50 (10.4)	3	80.1	3.7	0.9 (0.2-3.8)		
**Alcohol use**							
No	213 (44.2)	8	350.3	2.3	1.0	0.01	1.0
Yes	269 (55.8)	26	436.3	6.0	2.6 (1.2-5.8)		2.6 (1.1-6.0)
**Extramarital sex in past year**							
No	310 (64.3)	21	511.3	4.1	1.0	0.69	
Yes	172 (35.7)	13	275.4	4.7	1.1 (0.6-2.3)		
**Condom use frequency in past year**							
Did not use	193 (40.0)	23	308.2	7.5	1.0	0.001	1.0
Sometimes	210 (43.6)	10	344.3	2.9	0.4 (0.2-0.8)		0.4 (0.2-0.8)
Always	79 (16.4)	1	134.2	0.7	0.1 (0.01-0.7)		0.1 (0.02-0.9)
**Genital ulcer disease in past year**							
No	354 (73.4)	22	585.6	3.8	1.0	0.21	
Yes	128 (26.6)	12	201.0	6.0	1.6 (0.8-3.2)		
**Genital discharge in the past year**							
No	378 (78.4)	19	626.4	3.0	1.0	0.002	1.0
Yes	104 (21.6)	15	160.2	9.4	3.1 (1.6-6.1)		3.4 (1.6-7.2)
**Serological syphilis**							
Negative	457 (94.8)	31	745.9	4.2	1.0	0.37	
Positive	25 (5.2)	3	40.7	7.4	1.8 (0.5-5.9)		
**Receipt of ART by HIV-infected partner**							
Yes	80 (16.6)	3	138.6	2.2	1.0	0.33	
No	359 (74.5)	29	612.9	4.7	2.2 (0.7-7.2)		
Data not collected	43 (8.9)	2	35.1	5.7	2.6 (0.4-15.8)		

PYO, person-years of observation; RR, unadjusted rate ratio; aRR, adjusted rate ratio (All factors were adjusted for gender, age group at study entry, male circumcision, alcohol use, condom use frequency, and genital discharge in the past year); CI, confidence interval; LRT, likelihood ratio test.

Baseline factors that remained significantly associated with increased risk of HIV acquisition at multivariate analysis were young age [18–24 (aRR = 4.1, 95% CI, 1.6–10.8) and 25–34 (aRR = 2.7, 95% CI 1.2–5.8) years], alcohol use (aRR = 2.6, 95% CI 1.1–6), and reported genital discharge (aRR = 3.4, 95% CI: 1.6–7.2) in the past year. Condom use frequency in the year preceding enrolment was associated with a reduced risk of HIV acquisition [sometimes (aRR = 0.4, 95% CI 0.2–0.8); always (aRR = 0.1, 95% CI 0.02–0.9)]. Similar predictors of HIV acquisition were identified with Turnbull's exact interval censored maximum likelihood analysis method.

### Risk factors for HIV acquisition during study follow up

Crude and adjusted associations between risk factors present during study follow up and HIV incidence are presented in Table 2. At univariate analysis, factors that were significantly associated with increased risk of HIV acquisition during the study were young age [18–24 (RR = 10.0, 95% CI 3.7–26.9) and 25–34 (RR = 3.1, 95% CI 1.4–6.5) years], alcohol use (RR = 3.0, 95% CI 1.1–7.6), reported genital discharge (RR = 2.3, 95% CI 1.2–4.6), serological syphilis (RR = 2.7, 95% CI 1.2–6.1) and the HIV-infected partner being ART naïve (RR = 5.4, 95% CI 1.7–17.9). No seroconversions occurred among participants who reported always using condoms during the study. The risk of HIV acquisition was higher among participants that were uncircumcised (men), not married but with a steady partner, had genital ulcer disease, and those that reported alcohol use although these associations were not significant.

**Table 2:** Risk factors for incident HIV infection during follow up among HIV-uninfected partners in HIV discordant couples, Masaka, Uganda.

	N (%)	Seroconverters	PYO	Rate/100 PYO	RR (95% CI)	LRT p-value	aRR (95% CI)
**Total**	482 (100.0)	34	786.6	4.3			
**Male circumcision**							
Circumcised	94 (19.5)	3	154.3	1.9	1.0	0.20	1.0
Uncircumcised	236 (49.0)	20	384.6	5.2	2.7 (0.8-9.0)		2.7 (0.8-9.2)
Female	152 (31.5)	11	247.7	4.4	2.3 (0.6-8.2)		2.0 (0.5-7.7)
**Age group**							
≥35	293 (60.8)	11	504.0	2.2	1.0	0.001	1.0
25-34	166 (34.4)	17	255.0	6.7	3.1 (1.4-6.5)		2.3 (1.1-5.0)
18-24	23 (4.8)	6	27.6	21.7	10.0 (3.7-26.9)		6.2 (2.2-17.3)
**Education**							
≥Secondary	94 (19.5)	6	151.4	4.0	1.0	0.93	
Primary	338 (70.1)	25	555.2	4.5	1.1 (0.5-2.8)		
None	50 (10.4)	3	80.1	3.7	0.9 (0.2-3.8)		
**Religion**							
Non Muslim	383 (79.5)	30	622.6	4.8	1.0	0.16	
Muslim	99 (20.5)	4	164.0	2.4	0.5 (0.2-1.4)		
**Marital Status**							
Married	396 (82.2)	25	646.5	3.9	1.0	0.21	
Steady partner	86 (17.8)	9	140.1	6.4	1.7 (0.8-3.6)		
**Alcohol use**							
No	162 (33.6)	5	265.6	1.9	1.0	0.01	
Yes	320 (66.4)	29	521.1	5.6	3.0 (1.1-7.6)		
**Extramarital sex**							
No	270 (56.0)	19	445.2	4.3	1.0		
Yes	212 (44.0)	15	341.5	4.4	1.03 (0.5-2.0)	0.93	
**Condom use frequency**							
Sometimes	454 (94.2)	34	735.8	4.6	-	-	
Always	28 (5.8)	0	50.9	0.0	-	-	
**Reported genital ulcer**							
No	305 (63.3)	17	502.4	3.4	1.0	0.10	
Yes	177 (36.7)	17	284.2	6.0	1.8 (0.9-3.5)		
**Reported genital discharge**							
No	323 (67.0)	16	531.9	3.0	1.0	0.01	1.0
Yes	159 (33.0)	18	254.7	7.1	2.3 (1.2-4.6)		2.5 (1.1-5.5)
**Serological syphilis**							
Negative	438 (90.9)	27	717.0	3.8	1.0	0.04	1.0
Positive	44 (9.1)	7	69.6	10.1	2.7 (1.2-6.1)		3.2 (1.3-7.7)
**Receipt of ART by HIV-infected partner**							
Yes	152 (31.5)	3	270.6	1.1	1.0	0.002	1.0
No	287 (59.5)	29	480.9	6.0	5.4 (1.7-17.9)		4.8 (1.4-16.0)
Data not collected	43 (8.9)	2	35.1	5.7	5.1 (0.9-30.7)		3.9 (0.6-23.8)

PYO, person-years of observation; RR, unadjusted rate ratio; aRR, adjusted rate ratio (All factors were adjusted gender, age group at study exit, male circumcision, reported genital discharge, serological syphilis and ART use in the HIV-infected partner); CI, confidence interval; LRT, likelihood ratio test.

Factors that remained significantly associated with increased risk of HIV acquisition at multivariate analysis were young age [18–24 (aRR = 6.2; 95% CI: 2.2–17.3) and 25–34 (aRR = 2.3; 95% CI: 1.1–5.0) years], reported genital discharge (aRR = 2.5; 95% CI: 1.1–5.5), serological syphilis (aRR = 3.2; 95% CI: 1.3–7.7) and the HIV-infected partner being ART naïve (aRR = 4.8; 95% CI: 1.4–16.0). Similar predictors of HIV acquisition were identified with Turnbull's exact interval censored maximum likelihood analysis method.

## Discussion

The overall HIV incidence of 4.3 per 100 PYO in this cohort was high but lower than the rates reported in previous HIV discordant couple studies in Uganda [3], [14]. These previous studies were retrospective and conducted before ART became widely available in Uganda. Almost one third of the HIV-infected partners of our participants had initiated ART by the end of the study. Also, in our study, couples underwent HIV testing and risk reduction counseling, and were offered condoms at all study visits, which is likely to have resulted in some risk reduction. All these may help to explain the lower HIV incidence in our cohort compared to previous studies. Regular HIV testing and counseling is associated with increased condom use among couples [5], [15] and reduced heterosexual transmission of HIV in HIV discordant couples [15]. As discussed further below, our findings also confirm those by others that ART in the HIV-infected partner is associated with a decrease in heterosexual HIV transmission among discordant couples [16]. Although we provided information on the protective effect of male circumcision and referrals for those who wished to have the procedure, only one participant was circumcised during the study period. Male circumcision cannot therefore explain the relatively low HIV incidence observed in our study.

Also, participants who dropped out of the study were younger than those who completed it. This may have resulted in underestimation of HIV incidence since the risk of HIV acquisition was higher among younger participants. A higher HIV incidence among younger people has also been documented in previous studies [14]. This phenomenon could be due to cervical ectopy in younger women and an increase in minor genital trauma during intercourse among younger people [17], [18]. We did not collect data on frequency of sexual intercourse; however, frequency of intercourse in previous studies [17] did not explain the change in risk of HIV acquisition with age.

We found no significant difference in HIV incidence between men and women, the rate being about 4 per 100 PYO for each gender. Data on gender-specific HIV transmission risk from available literature is inconclusive. A study in the neighbouring district of Rakai found similar HIV transmission risks among men and women [14]. However, previous studies in Masaka [3] and Mwanza, Tanzania [4], found that HIV incidence in women with HIV-infected spouses was twice that of men. The reason for these conflicting results is unclear.

Alcohol use before sexual intercourse has been associated with increased risk of HIV acquisition in discordant couple [15] and in general population [19] cohorts. This is attributed to alcohol related disinhibition that results in increased risk behaviours such as non-use of condoms [15], [19]. Also, in vitro [20] and animal [21] studies have demonstrated that alcohol may enhance susceptibility to HIV infection through its effects on certain components of the immune system. In our study, a baseline history of alcohol use was associated with a nearly three-fold increase in the risk of HIV acquisition.

As expected, we found that regular condom use was strongly protective against HIV acquisition. Therefore, consistent condom use for prevention of HIV transmission among discordant couples in stable unions should be strongly promoted [15], [22]. However, our observation also confirms that in spite of the regular and intensive health education and condom promotion that we provided during the study, the acceptability and use of condoms in marriage or stable partnerships is low even among couples who are well informed about each other's HIV status. At least two reasons may be responsible for this: the fact that in Africa traditionally condoms have been associated with infidelity and prostitution and are therefore stigmatized [23]; and the strong desire of married couples to have children [5], [24].

Treatable RTIs are common and important correlates of HIV transmission within discordant heterosexual couples [25]. History of genital discharge and sero-syphilis diagnosis during the current study were each independently associated with an increased risk of HIV acquisition.

Though only borderline significant, the risk of HIV acquisition was higher among uncircumcised compared to circumcised men. Recent randomized clinical trials [26], [27] have provided compelling evidence that male circumcision significantly reduces the risk of HIV acquisition in heterosexual men. Once policies on male circumcision are in place and services rolled out, the effect of male circumcision on HIV incidence should be taken into account for sample size estimations in future HIV prevention trials. Even where such services are not available yet, it is likely to become an ethical condition that research projects will have to make them available.

We found that ART initiated according to the national HIV treatment guidelines [11] was associated with a reduced risk of heterosexual HIV acquisition. The risk of HIV acquisition was 6.0 per 100 PYO among participants whose HIV-infected partners were ART naïve, compared to 1.1 per 100 PYO among those whose partners were receiving ART, an 82% difference. Although earlier observational studies [16] had also reported a reduced risk of HIV transmission in the presence of ART, the evidence was insufficient to formulate policy and guidance on the role of ART in HIV prevention [28]. Conclusive evidence on the role of ART in HIV prevention has only become recently available following the early release of the HIV Prevention Trials Network (HPTN) 052 study results [29]. In this trial, early initiation of ART by HIV-infected individuals substantially protected their HIV-uninfected sexual partners from acquiring HIV infection, with a 96% reduction in risk of HIV transmission. Findings from this trial are expected to inform future recommendations on use of ART for HIV prevention among discordant couples [30]. Consequently, it may become an ethical requirement for HIV prevention research projects involving discordant couples to provide ART to the HIV-infected partners. It is likely that these developments will have an impact on study design and sample size of future HIV prevention trials; and such trials may become even more difficult should universal testing and ART treatment strategies be shown to be effective in reducing HIV transmission at the population level.

With the exception of ART, we did not collect data on other risk factors among HIV-infected partners that could facilitate HIV transmission. This was because the VPS protocol primarily targeted HIV-uninfected individuals at high risk of HIV acquisition. Early and late stage HIV infection, higher HIV load, genital ulcer disease, and younger age of the HIV-infected partner have all been associated with higher rates of HIV transmission [31].

Enrolment of participants in the current study overlapped that of the Microbicides Development Programme (MDP) 301 trial. MDP301 was a multicentre phase III trial of the PRO 2000/5 vaginal gel. At our centre, the trial recruited HIV-uninfected women who were in HIV discordant partnerships [32]. As a result, couples referred to and enrolled in current study were predominantly those in which the HIV-uninfected partner was male. We cannot determine the extent to which this gender bias affected the study results. Nonetheless, we believe that the findings may be applicable to other HIV discordant couple populations.

Our study has shown that in addition to being in an HIV discordant relationship, there are other important risk factors for HIV transmission among HIV discordant couples that need to be urgently targeted. The high HIV incidence and moderate participant retention observed in this study suggest that HIV-uninfected partners in discordant couples may be a suitable population for phase IIb/III preventive HIV vaccine trials in our setting. However, recent findings confirming that ART significantly reduces HIV transmission from an infected partner to an uninfected spouse may make it unfeasible to conduct future HIV prevention trials in this population.
